# Chiral PCB 91 and 149 Toxicity Testing in Embryo and Larvae (*Danio rerio*): Application of Targeted Metabolomics via UPLC-MS/MS

**DOI:** 10.1038/srep33481

**Published:** 2016-09-15

**Authors:** Tingting Chai, Feng Cui, Zhiqiang Yin, Yang Yang, Jing Qiu, Chengju Wang

**Affiliations:** 1 Institute of Quality Standards & Testing Technology for Agro-Products, Key Laboratory of Agro-product Quality and Safety, Chinese Academy of Agricultural Sciences, Key Laboratory of Agri-food Quality and Safety, Ministry of Agriculture, Beijing 100081, China; 2 College of Science, China Agricultural University, Beijing 100193, China

## Abstract

In this study, we aimed to investigate the dysfunction of zebrafish embryos and larvae induced by rac-/(+)-/(−)- PCB91 and rac-/(−)-/(+)- PCB149. UPLC-MS/MS (Ultra-performance liquid chromatography coupled with mass spectrometry) was employed to perform targeted metabolomics analysis, including the quantification of 22 amino acids and the semi-quantitation of 22 other metabolites. Stereoselective changes in target metabolites were observed in embryos and larvae after exposure to chiral PCB91 and PCB149, respectively. In addition, statistical analyses, including PCA and PLS-DA, combined with targeted metabolomics were conducted to identify the characteristic metabolites and the affected pathways. Most of the unique metabolites in embryos and larvae after PCB91/149 exposure were amino acids, and the affected pathways for zebrafish in the developmental stage were metabolic pathways. The stereoselective effects of PCB91/149 on the metabolic pathways of zebrafish embryos and larvae suggest that chiral PCB91/149 exposure has stereoselective toxicity on the developmental stages of zebrafish.

Although the production of PCBs ceased in many countries in the late 1970s, their extensive use in the past remains a serious environmental problem^1^. Recent studies have shown that many water sources have been polluted by different levels of PCBs^2^,^3^. Laboratory studies have demonstrated the toxicological effects of PCBs on aquatic ecosystems, including reproductive and immunological disorders^4^,^5^. With the wide recognition of chirality, it is a critical consideration for the accurate risk assessment of PCBs. A group of 19 PCB congeners contain a chiral axis and have two stable rotational enantiomers^6^, including PCB91 (2,2′,3,4′,6-pentachlorobiphenyl) and PCB149 (2,2′,3,4′,5′,6-hexachlorobiphenyl). In spite of the racemic (rac-) PCBs released into the environment, different processes are observed when isomers entered into organism^7^. Stereoselective biotransformation processes of chiral PCB91 and PCB149 are observed in a stream food web^8^. Non-racemic enrichment of chiral PCB91 is in predatory fish species^9^, but racemic enrichment of PCB149 in the juvenile rainbow trout^10^ and in Arctic char (*Salvelinus alpinus*)^11^ in laboratory experiments. Atropisomeric oxidation of chiral PCB91 and PCB149 is observed by live tissue in mice^12^. However, little research is about toxicological effects of chiral PCB91 and PCB149. Thus, it is essential to extend the stereoselective toxicology of chiral PCBs.

Zebrafish have been demonstrated to share many common features and molecular pathways with humans^13^, and zebrafish have emerged as an excellent model system for large-scale studies of vertebrate development and behaviour^14^. In the developmental stage, zebrafish have been employed as a model organism for toxicological studies^15^,^16^. Zebrafish embryos are sensitive to environmental changes^17^, easy to maintain and handle, and undergo rapid development^18^. Larvae hatch from their chorion 2–3 days post-fertilization and become free swimming with a rich repertoire of stereotyped motor behaviours, which operate on a simple blueprint of a vertebrate nervous system^19^.

Targeted metabolomics is defined as the measurement of defined groups of chemically characterized and biochemically annotated metabolites^20^. Based on previous studies of metabolic profiles, we found that organismal exposure to xenobiotics impacted the amino acids related to metabolism^21^,^22^,^23^. Amino acids are essential for the synthesis of proteins, polypeptides, and other molecules of biological importance and individual amino acids participate in and regulate key metabolic pathways related to reproduction, immunology, pathology, and cell biology^24^. For example, an imbalance of amino acids in an organism could result in metabolic diseases, including neurological dysfunction, and infectious diseases^25^.

During the last decade, metabolomics has been recognized as a powerful tool for investigating toxicity, including aquatic toxicology^26^. However, the studies on zebrafish embryos at metabolic levels is limited^27^,^28^. Ultra-performance liquid chromatography (UPLC) coupled with mass spectrometry (MS)-based multiple reaction monitoring (MRM) has been considered as the preferred technology to assessing targeted metabolomics^29^. This is the first report to clarify changes in zebrafish embryos and larvae after chiral PCB91 and PCB149 exposure using targeted metabolomics. The purpose of this study was to explore whether stereoselective disorders of amino acids and other metabolites occur in zebrafish embryos and larvae after rac-/(+)-/(−)- PCB91 and rac-/(−)-/(+)- PCB149. In addition, the characterized metabolic pathways were investigated through metabolomics combined with statistical analyses to explore dysfunction after PCB91/149 exposure. This information is intended to provide new insights into the assessment of the environmental risk of chiral PCBs in aquatic systems.

## Results

### Chemical analysis

Variations in the normal concentrations of rac-, (+)-, and (−)- PCB91 and PCB149 in the water were always below 20% during the day, suggesting that their concentrations remained constant in the exposed aquatic media.

### Quantification of amino acids in embryo and larvae

**Figure 1:**
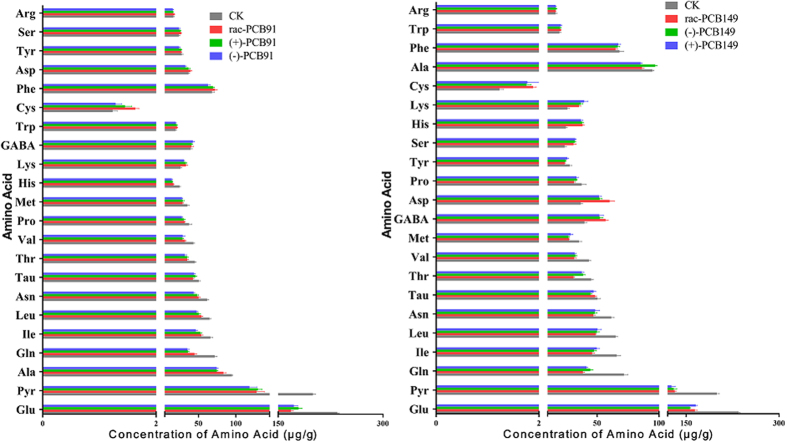
The amino acid content quantified in embryos after exposure to rac-/(+)-/(−)- PCB91 or rac-/(−)-/(+)- PCB149.

Supplementary Fig. S1 shows the mass chromatograms of the target amino acids, and the retention times for each amino acid are presented in Supplementary Table S1. The target amino acids included phenylalanine (Phe), tyrosine (Tyr), aspartic acid (Asp), glutamic acid (Glu), histidine (His), cysteine (Cys), Glutamine (Gln), methionine (Met), isoleucine (Ile), lysine (Lys), taurine (Tau), asparagine (Asn), γ-aminobutyric acid (GABA), pyroglutamic acid (Pyr), alanine (Ala), leucine (Leu), serine (Ser), threonine (Thr), arginine (Arg), valine (Val), proline (Pro) and tryptophan (Trp). Figure 1 shows the amino acid content in the embryo samples with the following characteristics: The Cys content was detected at relatively low levels (<2.00 μg/g), whereas the most abundant amino acids were Pyr and Glu at relatively high levels (>100.00 μg/g) in embryos after exposure to rac-/(+)-/(−)- PCB91 or PCB149. The levels of the other amino acids were in the range of 2.00–100.00 μg/g in embryos after exposure to rac-/(+)-/(−)- PCB91 or PCB149.

**Figure 2:**
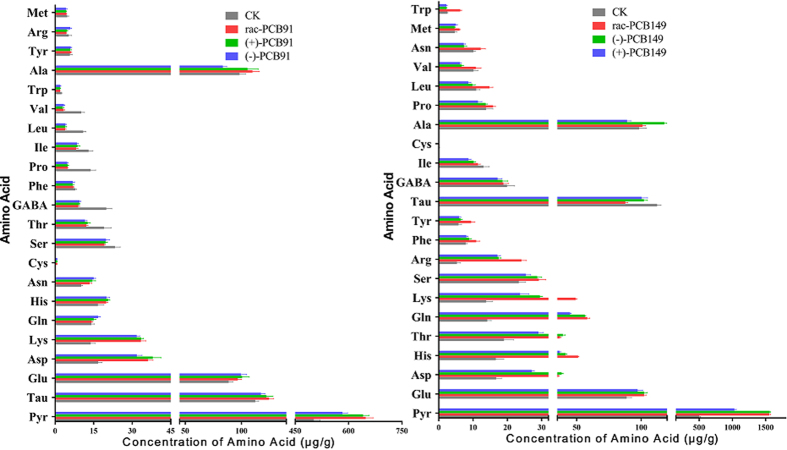
The amino acid content quantified in larvae after exposure to rac-/(+)-/(−)- PCB91 or rac-/(−)-/(+)- PCB149.

The results for the amino acid content in the larvae samples are shown in Fig. 2. The Cys content in the PCB91-treated larvae samples (0.70–0.90 μg/g) and PCB149-treated larvae samples (0.01–0.10 μg/g) was significantly different compared to that in the control groups (0.20 ± 0.05 μg/g). In the larvae, the most abundant amino acids were Ala, Glu, and Tau, with concentrations higher than 100.00 μg/g, and Pyr. The concentrations of Pyr in the PCB91-treated groups were 648.31 ± 20.52 μg/g after rac-PCB91 exposure, 640.99 ± 16.51 μg/g after (+)-PCB91 exposure, and 582.75 ± 14.52 μg/g after (−)-PCB91 exposure, all higher than in the control groups (502.45 ± 17.46 μg/g). The concentrations of Pyr in the PCB149-treated groups were 1553.58 ± 20.55 μg/g after rac-PCB91 exposure, 1560.58 ± 13.94 μg/g after (−)-PCB149 exposure, and 1029.83 ± 28.07 μg/g after (+)-PCB149 exposure.

### Semi-quantitation of other metabolites in embryo and larvae

A total of 22 metabolites selected from the MassBank (www.massbank.jp) database were detected in the embryo or larvae samples. The 22 metabolites in our study were selected via UPLC-MS/MS analysis. If a metabolite exhibited one or two product ion (Q_3_) without a peak, the metabolite was eliminated. Additionally, if a metabolite presented two Q_3_ that showed unmatched retention times, then this metabolite was eliminated. If one metabolites had more than two Q_3_, we checked the Q_3_ mass spectra and the isotopic peaks compared the mass spectra of the two Q_3_ mass spectra using the MassBank database; All of the metabolites for which the mass spectra of the two Q_3_ mass spectra exhibited incorrect peak ratios were also eliminated. The details of these metabolites are listed in Supplementary Table S2. The levels of these metabolites in the treated groups of embryos (Table 1) and larvae (Table 2) are presented as increases or decreases in the area ratio (%) compared with the control groups.

As shown in Table 1, the urea and betaine contents in the embryos were not significantly affected after exposure to rac-/(+)-/(−)- PCB91; however, they were significantly decreased in embryos after exposure to rac-/(−)-/(+)- PCB149. Furthermore, there was a significant increase in saccharopine after rac-/(−)-/(+)- PCB149 exposure but no change after rac-/(+)-/(−)- PCB91 exposure. The 5-aminolevulinic acid content was decreased by approximately 21.19–27.44% in the rac-/(+)-/(−)- PCB91-treated groups and was decreased by approximately 66.43–73.27% in the rac-/(−)-/(+)- PCB149-treated groups. Although the 5-aminolevulinic acid content was decreased after exposure to rac-/(+)-/(−)- PCB91 or PCB149, the degree of the reduction differed.

According to Table 2, there was no obvious change in the dimethylglycine, betaine, and 2-aminolevulinic acid content in larvae after PCB91 and 149 exposures. For example, the uridine content was almost invariant after rac-PCB91 exposure, decreased by approximately 36.61% after (+)-PCB91 exposure, decreased by approximately 48.60% after (−)-PCB91 exposure, almost invariant after rac-PCB149 exposure, increased by approximately 35.17% after (−)-PCB149 exposure, and increased by approximately 53.73% after (+)-PCB149 exposure.

### Chemometrics

**Figure 3:**
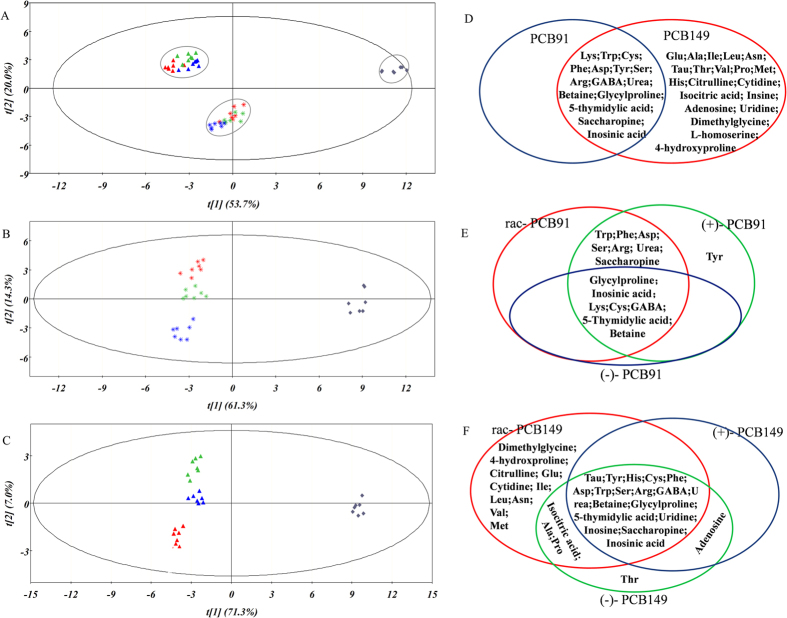
Multivariate analysis of the target metabolites in embryos after exposure to rac-/(+)-/(−)- PCB91 or rac-/(−)-/(+)- PCB149. (**A**) PCA scores plotted for all treated samples; (**B**) PLS-DA scores plotted for rac-/(+)-/(−)- PCB91-treated samples; (**C**) PLS-DA scores plotted for rac-/(−)-/(+)- PCB149-treated samples; (**C**) Venn plot for the characteristic metabolites of PCB91/149-treated samples; (**D**) Venn plot for the characteristic metabolites of rac-/(+)-/(−)- PCB91-treated samples; and (**F**) Venn plot for the characteristic metabolites of rac-/(+)-/(−)- PCB91-treated samples. The squares, stars and triangles correspond to the control, PCB91- and PCB149-treated groups, respectively. The red, green, and blue colours correspond to the rac-/(+)-/(−)- PCB91, respectively, in plot (**B**). The red, green, and blue colours correspond to rac-/(−)-/(+)- PCB149, respectively, in plot (**C**).

The PCA analysis based on the metabolite content in embryos is shown in Fig. 3. The PCA parameters for the explained variation (R^2^) and the cross-validated predictive ability (Q^2^) were 82.0% and 71.3%, respectively. The first two PCA components (t[1] and t[2]) explained 73.7% of the variation in the metabolite data, showing the split of the seven treated groups into three individual groups (Fig. 3A). The PLS-DA parameters for R^2^ and Q^2^ were 87.4% and 75.7% for the PCB91-treated embryo groups, respectively, and 91.8% and 84.7% for the PCB149-treated embryo groups, respectively. The first two PLS-DA components explained 75.6% of the variation in the metabolite data of the PCB91-treated embryo groups (Fig. 3B) and 78.3% of the variation in the metabolite data of the PCB149-treated embryo groups (Fig. 3C). The relationship of the characteristic metabolites between the PCB91-treated embryos and the PCB149-treated embryos is presented in Fig. 3D. The relationship of the characteristic metabolites in rac-/(+)-/(−)- PCB91-treated embryos is presented in Fig. 3E, and that of the rac-/(−)-/(+)- PCB149-treated embryos is presented in Fig. 3F.

**Figure 4:**
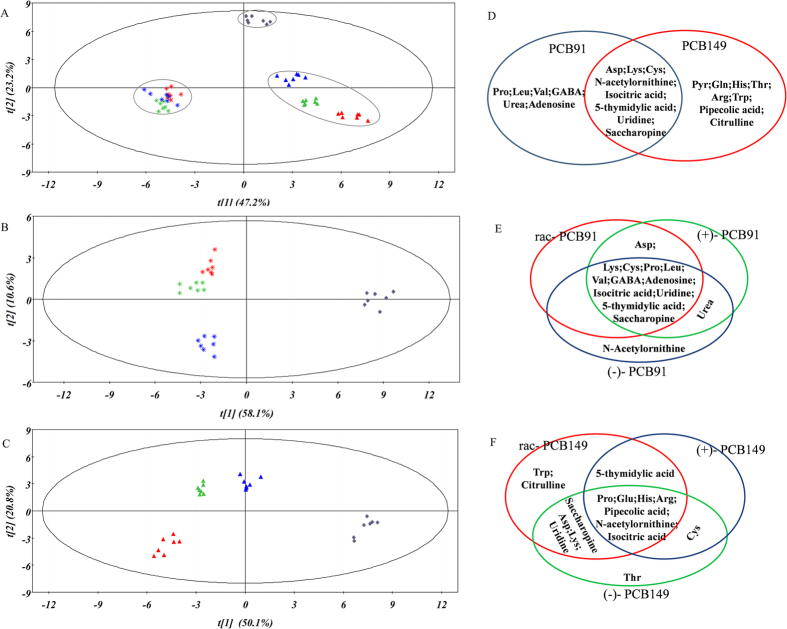
Multivariate analysis of the target metabolites in larvae after rac-/(+)-/(−)- PCB91 or rac-/(−)-/(+)- PCB149 exposure. (**A**) PCA scores plotted for all treated samples; (**B**) PLS-DA scores plotted for rac-/(+)-/(−)- PCB91-treated samples; (**C**) PLS-DA scores plotted for rac-/(−)-/(+)- PCB149-treated samples; (**C**) Venn plot for the characteristic metabolites for PCB91/149-treated samples; (**D**) Venn plot for the characteristic metabolites for rac-/(+)-/(−)- PCB91-treated samples; (**F**) Venn plot for the characteristic metabolites for rac-/(+)-/(−)- PCB91-treated samples. The squares, stars and triangles correspond to the control, PCB91- and PCB149-treated groups, respectively. The red, green, and blue colours correspond to rac-/(+)-/(−)- PCB91, respectively, in plot (**B**). The red, green, and blue colours correspond to rac-/(−)-/(+)- PCB149, respectively, in plot (**C**).

The PCA plot for the metabolites in treated larvae is presented in Fig. 4A, and the PCA parameters for R^2^ and Q^2^ were 95.7% and 87.5%, respectively. The first two PCA components explained 70.4% of the variation in the metabolite data. The first two PLS-DA components showed the split of the rac-/(+)-/(−)- PCB91-treated groups plus the control group into individual groups (Fig. 4B) and the split of the rac-/(−)-/(+)- PCB149-treated groups plus the control group into individual groups (Fig. 4C). The PLS-DA parameters for R^2^ and Q^2^ were 95.4% and 52.7% for the PCB91-treated larvae groups, respectively, and 94.5% and 95.8% for the PCB149-treated larvae groups, respectively. The first two PLS-DA components explained 68.7% of the variation in the metabolite data of the PCB91-treated larvae groups and 70.9% of the variation in the PCB149-treated larvae groups. The relationship of the characteristic metabolites between the PCB91-treated embryos and the PCB149-treated embryos is presented in Fig. 4D. The relationship of the characteristic metabolites in the rac-/(+)-/(−)- PCB91-treated embryos is presented in Fig. 4E, and the data for the rac-/(−)-/(+)- PCB149-treated embryos are presented in Fig. 4F.

## Discussion

Metabolomics in combination with PCA and PLS-DA enables us to not only detect the targeted metabolites but also to discover unique metabolites and thus generate new hypotheses because the change in the metabolites is directly combined with the phenotype^30^. According to the results in Figs 3 and 4, most of the unique metabolites in the embryos and larvae after PCB91/149 exposure were amino acids. To explore the possible pathways affected by PCB91 and 149, the characteristic metabolites were shown in Figs 3D and 4D.

The metabolic pathways affected by chiral PCB91 and PCB149 could explain their toxic metabolism in embryo and larvae. Metabolic pathway analysis was performed in characteristic metabolites significantly altered during PCB91/149 exposure, as shown in Table 3. The common pathways observed in the embryos and larvae were Arg and Pro metabolism and Ala, Asp and Glu metabolism. Increasing evidence shows that the two metabolic pathways participate in the genetic and epigenetic regulation of cell growth and development. For example, Arg, and Pro are essential for DNA and protein synthesis and participate in protein and DNA methylation, Gln is a major energy substrate for rapidly dividing cells, and Gln and Asp are major metabolic fuels^31^. Given that energy is the currency of the developed stages of zebrafish, the ability of embryo and larvae to acquire, transform, store and efficiently use energy is essential for their survival^32^. A previous study has demonstrated that increasing concentrations of PCB126 in rainbow trout could decrease tissue fuel supplies and metabolic rates^33^, which is consistent with our results. In the present study, the levels of these amino acids were altered, indicating that PCB91/149 could affect the growth and development of embryo and larvae.

Furthermore, previous studies have demonstrated that exposure to PCBs could cause oxidative damage to organisms at the enzyme and gene expression levels. Antioxidative enzyme activities declined after zebrafish embryos were exposed to PCB126^34^. The exposure of zebrafish embryos to PCB126 led to the induction of antioxidative gene expression^35^. The mRNA expression levels of antioxidant genes in rare minnow larvae were remarkably higher after exposure to high concentrations of Aroclor1254^36^. In our previous studies, PCB149 caused the abnormal expression of antioxidative genes in embryo-larvae^37^ and adult zebrafish^38^. In the present study, these metabolites were found to be related to antioxidant progress. Arg inhibited the expression of pro-oxidative and lipogenic genes related to antioxidative responses in a cell-specific manner; Pro, citrulline, and N-acetylornithine were also considered antioxidants^39^; and Gln is essential for the expression of anti-oxidative genes^40^. Moreover, persistently high levels of Pyr could be considered an indicator of increased oxidative stress^41^. In our study, the level of Pyr was significantly elevated in larvae after PCB91/149 exposure. The elevated levels of Pyr could have been caused by the following process: Gln depletion stimulates the production of γ-glutamyl Cys from Cys and Glu and the high levels of γ-glutamyl Cys are then converted to Pyr. The change in the levels of these metabolites in embryos and larvae after PCB91/149 exposure also demonstrated that exposure to PCBs is associated with cytotoxic effects in organisms, exceeding antioxidant cell defences, at the metabolic level.

The characteristic pathways observed in embryos after PCB91/149 exposure were Phe, Tyr and Trp biosynthesis and Gly, Ser and Thr metabolism. These two metabolic pathways participate in neurodevelopment. Phe regulates neurological development and function; Trp and Tyr have an important role in the functioning of neurotransmitters such as dopamine and nor-dopamine; and dimethylglycine is a derivative of the amino acid glycine, which is a neurotransmitter in the central nervous system^39^. Thr is used to treat various nervous system disorders^42^. Ser also functions as a novel neurotransmitter^43^. In addition, Arg is considered as the precursor for nitric oxide biosynthesis, which plays important roles in neurotransmission^44^. Gln is also a neurotransmitter in the central nervous system^45^. GABA is the chief inhibitory neurotransmitter in the central nervous system and plays a principal role in reducing excitability throughout the nervous system. PCB exposure has been associated with neurodegenerative diseases, such as Parkinson’s disease, amyotrophic lateral sclerosis, and dementia^46^. A genetic neurodevelopmental disorder was observed in post-mortem brain samples from individuals with higher levels of PCB95^47^. Experimental studies also have implicated multiple ortho-substituted PCB congeners in developmental neurotoxicity^48^. PCB153 induced neostriatal toxicity in adult WKY and SHR rats through changes in the monoaminergic but not amino acidergic neurotransmitter systems^49^. The effect of exposure of zebrafish larvae to a PCB mixture and Aroclor 1254 on neurodevelopment was also observed^50^. Thus, the change in these amino acids in our studies underlined the nerve-endocrine dysfunction in embryos after PCB91/149 exposure.

The characteristic pathways observed in embryos after PCB149 exposure were Val, Leu and Ile biosynthesis and Cys and Met metabolism. Val, Leu and Ile biosynthesis was observed in larvae after PCB91 exposure. Leu and Val are branched chain amino acids (BCAAs), and an appropriate ratio of BCAAs can prevent amino acid imbalance^51^. BCAAs are important amino acids in energy metabolism, and changes in BCAA levels can result in the disturbance of three major nutrients: sugar, fat and protein. Cys can be synthesized from Met in the liver^52^, and Cys and Met also participate in cellular metabolism and nutrition.

The stereoselective effects of chiral PCB91 and PCB149 exposure on metabolic pathways were also investigated in embryo and larvae. As shown in Table 4, non-racemic effects were observed in the embryo after exposure to chiral PCB91 and PCB149, whereas racemic effects were observed in the larvae after exposure to these PCBs. Rac- and (+)- PCB91 exposure had obvious effects on the metabolic pathways related to cell growth and antioxidant defences. However, these metabolic pathways were not obviously affected in the embryos after (−)-PCB91 exposure. Common toxic effects were observed on cell growth and neurodevelopment, and the toxic effects on energy metabolism were also observed in the embryos after rac-PCB149 exposure. Significant differences in toxic effects were not observed in the larvae at metabolic levels after rac- and (−)- PCB149 exposure. This finding is consistent with the dysregulation of gene expression observed in the embryos and larvae after exposure to rac- and (−)-PCB149 37. The metabolic pathway affected by PCB149 in embryo suggested that synergistic effects might occur when two isomers of PCB149 coexisted. In our previous study, we demonstrated that synergistic effects occurred after prolonged exposure of zebrafish to rac-PCB149. For example, additional dysregulation of gene expression related to inflammation-associated diseases were observed in zebrafish embryo-larvae^37^ and adult zebrafish^38^ after rac-PCB149 exposure. The dysregulation of antioxidant gene expression was in embryo-larvae after racemic PCB149 exposure^37^, then after (+)-, and (−)- PCB149 exposure. Chiral PCB91 and PCB149 induced antioxidant defences in zebrafish. For example, chiral PCB149 may stereoselectively induce antioxidant defences in zebrafish embryo-larvae and adult zebrafish at gene expression level^37^,^38^. PCB91 stereoselectively induced oxidative stress by altering the reactive oxygen species levels, malondialdehyde contents, antioxidant enzyme activities and gene expressions in the brain and liver tissues of adult zebrafish^53^. Previous studies also found that chiral PCBs induced stereoselective toxicity. For example, PCB84 atropisomers stereoselectively affected [^3^H] phorbol ester binding in rat cerebellar cells, indicating atropselectively neurodevelopmental toxicity^54^. (−)-PCB136 enhanced the binding of [^3^H]ryanodine to high-affinity sites on ryanodine receptors type 1 and type 2, whereas (+)-PCB136 did not have an effect on [^3^H]ryanodine binding, which suggested stereoselective toxicological impacts on Ca^2+^ channels^55^.

In conclusion, the profiles of various metabolite levels in embryos and larvae zebrafish after chiral PCB91/149 exposure indicated that PCB91/149 exposure is a risk factor for aquatic organisms. Therefore, further research on the risk PCBs pose to aquatic organisms should be performed to clarify the negative impact of PCBs on the environment.

## Experimental Section

### Zebrafish husbandry

Juvenile AB strain zebrafish (*Danio rerio*), obtained from the Beijing Hongdagaofeng Aquarium Department, were cultured in the fish facility (Esen Corp.) at 26 °C with a photoperiod of 14/10 h (light/dark). The fish were fed dried brine shrimp (equivalent to 2% of the fish body weight) daily. The preparation and collection of the zebrafish embryos followed previously described methods^56^. Healthy developing embryos were identified under a microscope within 2 h of natural spawning of healthy adults and were grown in embryo medium.

### Chemicals and reagents

Rac- PCB149 (99.9%) and PCB91 (99.9%) were purchased from Dr. Ehrenstorfer GmbH (Germany). The separation of racemate and the quantification of the isomers were conducted according to a previously published procedure^38^,^57^. The purities of (−)-/(+)- PCB149 and (+)-/(−)- PCB91 were higher than 98.0%. The rac-/(−)-/(+)- PCB149 and PCB91 were dissolved in acetone for the exposure experiments. All the standard amino acids were purchased from the National Institute of Metrology (China).

A standard solution containing 2 mM Ca^2+^, 0.5 mM Mg^2+^, 0.75 mM Na^+^ and 0.074 mM K^+^ was used for the following tests. All organic reagents in this study were of HPLC grade, and the other reagents were of analytical grade.

### Exposure and sample collection

This study conformed with Chinese legislation and was approved by the independent animal ethics committee at China Agricultural University. During exposure experiments, conditions, including temperature, humidity and light cycle, were the same as the culture environment.

Seven 500 mL beakers with 200 mL standard solution were prepared for each dose treatment. Each beaker contained sixty healthy embryos, considered as one repetition and thus each dose treatment contained seven repetitions. The dose treatments were designed as follows: rac- PCB149 at 1 μg/L, (−)-PCB149 at 1 μg/L, (+)-PCB149 at 1 μg/L, rac- PCB91 at 1 μg/L, (−)-PCB91 at 1 μg/L, (+)-PCB91 at 1 μg/L, plus solvent control. The standard solution with acetone was used as the solvent control, and concentration of acetone in the test solutions was less than 0.01% (v/v).

Fifteen embryos were randomly collected from each beaker at the hatching point (3 days post-fertilization) and yolk sac disappearing point (7 days post-fertilization). The collected samples were stored at −80 °C until extraction. During the exposure period, the death of fish embryos less than three only occurred in one or two replicates. The lethality was less than 1% in total seven replication of each treated groups. Then few dead embryos were removed immediately. The exposure medium was renewed every 24 h.

### Metabolite extraction

Metabolites were extracted from whole embryos using the two-step methanol:water:chloroform (2:1.8:2 final solvent ratio) protocol as described previously^58^. Briefly, pre-weighed frozen samples were homogenized in 8 μL/mg cold methanol and 3.2 μL/mg cold water using an electric homogenizer (Tiangen Biotech, China). All of the remaining solvents (8 μL/mg chloroform and 4 μL/mg water) were added to the homogenates. The samples were vortexed for 1 min, left on ice for 10 min to partition and then centrifuged for 20 min at 12,000 rpm at 4 °C. The upper layers were diluted 10-fold and then transferred into 1.5 mL auto-sampler vials.

### UPLC-MS/MS analysis

Quality control (QC) samples was prepared by mixing equal volumes (15 μL) from each sample. The QC sample was used to estimate a “mean” profile that represented all the encountered analytes^59^.

UPLC analysis was performed on Xbridge C18 columns (4.6 mm × 150 mm, 3.5 μm; Waters, USA) using an ACQUITY UPLC (Waters, USA). The separation program was maintained at 25 °C with a flow rate of 0.3 mL/min. The mobile phase consisted of solvent A, i.e., 0.1% formic acid in water, and solvent B, i.e., acetonitrile. The gradient duration program was as follows: 0–2 min, 5% B; 2–10 min, 5–60% B; 10–13 min, 60–5% B; and 13–16 min, 5% B.

MS/MS analysis was conducted with a QTrap 6500 mass spectrometer (AB SCIEX, USA). Data acquisition and processing were performed with Analyst 1.6.2 software. MS/MS detection was performed in the MRM in the positive electrospray ionization (ESI+) mode. After optimization of the instrumental parameters, gas 1 (GS 1) and gas 2 (GS 2) were set at 60 and 65 psi, respectively. The spray voltage, collision exit potential and collision entrance potential were +5500, 10 and 8 V, respectively. Other parameters, including the declustering potential (DP), collision energy (CE), precursor ion (Q1) and product ion (Q3), for each amino acid were optimized and are presented in Supplementary Table S1.

### Methodology

The reliability of the preparation method was evaluated by measuring the recoveries of targeted amino acids. The spiked samples with external standard at three concentration levels (2, 50, and 500 μg/g) for each amino acid were detected in the embryos/larvae and determined with calibration curves. Recovery was expressed as [(found concentration −basic concentration)/spiked concentration] × 100%. The average recoveries for all amino acids ranged from 87.6% to 118.0% for the embryo samples and from 92.4% to 113.0% for the larva samples.

The amino acids were quantified using the external standards based on peak areas of the mass chromatogram. The calibration curves showed good linearity in the range of 0–500 μg/L with correlation coefficients of 0.9903 or greater for each amino acid.

The intra-day (n = 7) and inter-day (3 days) precisions, expressed as relative standard deviation (%RSD) of QC samples, were calculated to evaluate the reproducibility of the analytical samples. The RSD values were in range of 4.19–14.17% for the embryo samples and in the range of 2.46–18.20% for the larva samples. The linearity, precision and recovery results are summarized in Supplementary Table S3. The results clearly indicated that the analyses under the defined conditions were reliable and stable for the exploration of the information contained in the biological system.

### Statistical analysis

Statistical analyses were performed using SPSS16.0 software. The data on the metabolite content were imported into SIMCA-P software (Umetrics, Sweden) for principal component analysis (PCA) and partial least squares-discriminate analysis (PLS-DA). The characteristic metabolites of treated groups in Venn plot were obtained using MPP (Mass Profiler Professional) software (Agilent, USA) through statistical analysis including *t*-tests (p ≤ 0.05), multiple testing correction (Benjamini Hochberg FDR) and fold-change (≥2). The characteristic metabolites were imported into MetaboAnalyst (www.metaboanalyst.ca) and MBrole (csbg.cnb.csic.es/mbrole). The characteristic pathways affected by chiral PCB91 and PCB149 were obtained with p value less than 0.05.

## Additional Information

**How to cite this article**: Chai, T. *et al.* Chiral PCB 91 and 149 Toxicity Testing in Embryo and Larvae (*Danio rerio*): Application of Targeted Metabolomics via UPLC-MS/MS. *Sci. Rep.* **6**, 33481; doi: 10.1038/srep33481 (2016).

## Supplementary Material

Supplementary Information

## Figures and Tables

**Table 1:** The characteristic metabolite content in embryos.

Metabolite	PCB91			PCB149		
	rac-	(+)-	(−)-	rac-	(−)-	(+)-
Urea	—	—	—	↓↓↓↓	↓↓↓↓	↓↓↓↓
Dimethylglycine	↓	↓	↓	↓	↓	↓
Betaine	—	—	—	↓↓↓↓	↓↓↓↓	↓↓↓↓
L-Homoserine	↓	↓	↓	↓↓	↓	↓
Pipecolic acid	↓	↓	↓	↓	↓	↓
5-Aminolevulinic acid	↓	↓	↓	↓↓↓	↓↓↓	↓↓↓
4-Hydroxyproline	—	—	↓	↓	↓	↓
Creatine	↓↓	↓↓	↓↓	↓↓	↓↓	↓↓
2-Aminobenzoic acid	↓	↓	↓↓	↓↓	↓↓	↓↓
Xanthine	↓↓	↓↓	↓↓	↓↓	↓↓	↓↓
Aminoadipic acid	↓↓	↓↓	↓↓	↓↓	↓↓	↓↓
Glycylproline	↑↑↑↑	↑↑	↑	↑	↑↑	↑
Citrulline	↓	↓	↓	↓	↓	↓
Isocitric acid	↓	↓	↓	↓	—	↓
5-Thymidylic acid	↑↑↑↑	↑↑↑↑	↑↑↑	↑↑↑	↑↑↑↑	↑↑↑↑
Cytidine	↓↓↓	↓↓↓	↓↓	↓↓↓↓	↓↓↓	↓↓↓
Uridine	↓↓	↓↓	↓↓	↓↓↓	↓↓↓	↓↓
Adenosine	↓↓	↓	↓	↓↓	—	—
Inosine	↓↓	↓↓↓	↓↓↓	↓↓↓↓	↓↓↓↓	↓↓↓↓
Saccharopine	—	—	—	↑↑↑↑	↑↑↑↑	↑↑↑↑
Inosinic acid	↓↓↓↓	↓↓↓↓	↓↓↓↓	↓↓↓↓	↓↓↓↓	↓↓↓↓

Increased or decreased area ratio (%): 0–20%, -; 20–40%, ↓/↑; 40–60%, ↓↓/↑↑; 60–80%, ↓↓↓/↑↑↑; >80%, ↓↓↓↓/↑↑↑↑.

**Table 2:** The characteristic metabolite content in larvae.

Metabolite	PCB91			PCB149		
	rac-	(+)-	(−)-	rac-	(−)-	(+)-
Urea	↓	↓↓	↓↓↓	—	—	—
Dimethylglycine	—	—	—	—	—	—
Betaine	—	—	—	—	—	—
L-Homoserine	↓	↓	↓	↑↑↑↑	↑↑↑	↑↑
Pipecolic acid	↑↑↑	↑↑↑	↑↑↑	↑↑↑↑	↑↑↑↑	↑↑↑↑
5-Aminolevulinic acid	—	↑	—	—	—	—
4-Hydroxyproline	↓↓	↑↑	↓	—	↓	↓
Creatine	—	↑	—	—	—	—
2-Aminobenzoic acid	—	—	—	—	—	—
Xanthine	↑↑	↑↑	↑↑	—	—	—
Aminoadipic acid	↑↑↑	↑↑	↑	↑↑↑	↑↑↑	↑↑
Glycylproline	↓	—	↓	↑	↑	↑
Citrulline	↑↑↑	↑↑↑↑	↑↑↑↑	↑↑↑↑	↑↑↑↑	↑↑↑↑
Isocitric acid	↓	—	↑	↑↑↑↑	↑↑↑	↑↑
5-Thymidylic acid	↑↑↑↑	↑↑↑↑	↑↑↑↑	↑↑↑↑	↑↑↑↑	↑↑↑↑
Cytidine	↓↓↓	↓↓↓	↓↓↓	↓↓	↓↓	↓↓
Uridine	—	↓	↓↓	—	↑	↑↑
Adenosine	↓↓↓	↓↓↓	↓↓↓	↓↓↓	↓↓↓	↓↓
Inosine	↓↓	↓↓↓	↓↓↓↓	—	—	—
Saccharopine	↑	↑↑	↑	—	—	—
Inosinic acid	↓↓↓↓	↓↓↓↓	↓↓↓↓	↓↓↓	↓↓↓↓	↓

Increased or decreased area ratio (%): 0–20%, -; 20–40%, ↓/↑; 40–60%, ↓↓/↑↑; 60–80%, ↓↓↓/↑↑↑; >80%, ↓↓↓↓/↑↑↑↑.

**Table 3:** The characteristic pathways in embryo and larvae induced by PCB91 and PCB149.

Pathway	Embryo				Larvae			
	PCB91		PCB149		PCB91		PCB149	
	p	Compound	p	Compound	p	Compound	p	Compound
Arg and Pro metabolism	0.0088	Urea; Arg GABA	5.27 × 10^−4^	Pro; GABA; Arg; 4-Hydroxyproline; Citrulline; Urea	7.32 × 10^−4^	GABA; Pro; Urea; Arg	0.0013	Arg; Gln; Citrulline; N-Acetylornithine;
Ala, Asp and Glu metabolism	0.0246	GABA; Asp	0.0210	Asn; GABA; Asp;	0.0246	GABA; Asp	0.0317	Asp; Gln
Phe, Tyr and Trp biosynthesis	6.02 × 10^−4^	Phe; Tyr;	0.0036	Tyr; Phe;				
Gly, Ser and Thr metabolism	0.0397	Betaine; Ser;	0.0067	Dimethylglycine; Betaine; Thr; Ser; L-Homoserine;				
Val, Leu and Ile biosynthesis			0.0036	Thr; Val; Leu;	0.0074	Leu; Val		
Cys and Met metabolism			0.0348	Met; Cys; Ser; L-Homoserine;				

**Table 4:** The characteristic pathways in embryo and larvae induced by rac-/(+)-/(−)- PCB91 and PCB149.

	Embryo						Larvae					
	PCB91			PCB149			PCB91			PCB149		
	rac-	(+)-	(−)-	rac-	(+)-	(−)-	rac-	(+)-	(−)-	rac-	(+)-	(−)-
Arg and Pro metabolism	√	√	—	√	√	√	√	√	√	√	√	√
Ala, Asp and Glu metabolism	√	√	—	√	—	—	√	√	√	√	—	√
Phe, Tyr and Trp biosynthesis	√	√	—	√	√	√	—	—	—	—	—	—
Gly, Ser and Thr metabolism	√	√	—	√	—	√	—	—	—	—	—	—
Val, Leu and Ile biosynthesis	—	—	—	√	—	—	√	√	√	—	—	—
Cys and Met metabolism	—	—	—	√	—	—	—	—	—	—	—	—

“√” indicates the metabolic pathway affected after PCBs exposure (p < 0.05).
