# Supplementary material for: Chiral PCB 91 and 149 Toxicity Testing in Embryo and Larvae (Danio rerio): Application of Targeted Metabolomics via UPLC-MS/MS
Source: Sci Rep. 2016 Sep 15;6:33481. doi: 10.1038/srep33481 (PMC5024159; doi:10.1038/srep33481)
Supplement: Supplementary Information [file srep33481-s1.pdf]

Chiral PCB 91 and 149 Toxicity Testing in Embryo and Larvae (*Danio rerio*): Application of Targeted Metabolomics via UPLC-MS/MS

Tingting Chai[1,2], Feng Cui[2], Zhiqiang Yin[1], Yang Yang[2], Jing Qiu[1*] & Chengju Wang[2*]

[1] Institute of Quality Standards & Testing Technology for Agro-Products, Key Laboratory of Agro-product Quality and Safety, Chinese Academy of Agricultural Sciences; Key Laboratory of Agri-food Quality and Safety, Ministry of Agriculture, Beijing 100081, China

[2] College of Science, China Agricultural University, Beijing 100193, China

Fig. S1 UPLC-MS/MS chromatograms of target amino acids.

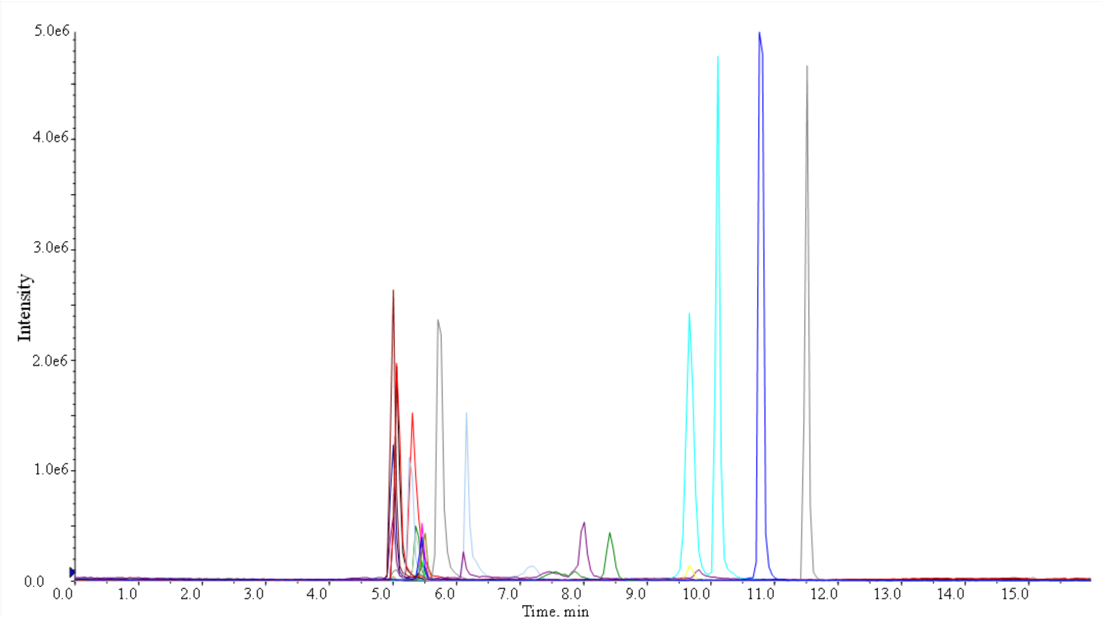

Table S1. Multiple reaction monitoring conditions for the analysis of target amino acids.

Amino acid	Q1	Q3	DP (V)	CE (V)	Retation time(min)
Phenylalanine	166.09	120.08*	60	20	10.79
(Phe)		103.06	50	35	
Tyrosine	182.08	136.08*	60	20	8.41
(Tyr)		165.05	40	15	
Aspartic acid	134.05	88.04*	80	15	5.43
(Asp)		116.04	120	10	
Glutamic acid	148.20	84.10*	50	20	5.50
(Glu)		102.00	50	15	
Histidine	156.01	110.07*	80	20	5.06
(His)		93.05	60	30	
Cystine	241.03	151.98*	60	20	5.27
(Cys)		120.01	50	25	
Glutamine	147.08	130.05*	15	15	5.00
(Gln)		84.05	20	20	
Methionine	150.20	104.00*	50	15	7.77
(Met)		133.00	30	15	
Isoleucine	132.10	69.07*	80	30	9.66
(Ile)		86.20	80	30	
Lysine	147.10	84.10*	15	20	5.00
(Lys)		130.00	15	15	
Taurine	126.02	108.01*	40	15	5.46
(Tau)		83.20	120	15	
Asparagine	133.06	87.06*	15	10	5.36
(Asn)		74.02	15	20	
γ-aminobutyric acid	104.07	58.07*	60	40	5.31
(GABA)		87.04	15	15	
Pyroglutamic acid	130.05	84.05*	60	15	5.00
(Pyr)		112.90	15	15	
Alanine (Ala)	90.06	44.05*	50	15	5.45
Leucine (Leu)	132.10	86.10*	60	15	10.11
Serine (Ser)	106.10	60.00*	60	15	5.37
Threonine (Thr)	120.10	74.10*	50	15	5.46
Arginine (Arg)	175.20	70.00*	80	25	5.07
Valine (Val)	118.10	72.20*	60	15	6.16
Proline (Pro)	116.07	70.00	60	20	5.73
Tryptophan (Trp)	205.20	188.30*	60	15	11.51

*quantitative ion

Table S2. Multiple reaction monitoring conditions for metabolites.

Metabolites	Q1	Q3		Retation time(min)
Urea	61.04	44.01*		5.63
Dimethylglycine	104.00	86.20*	58.10	5.36
Betaine	118.09	58.10*	59.07	5.51
L-Homoserine	120.10	74.00*	56.20	5.41
Pipecolic acid	130.10	84.10*	56.20	5.35
5-Aminolevulinic acid	132.00	114.00*	86.20	5.56
4-Hydroxyproline	132.07	86.06*	60.50	9.92
Creatine	132.08	90.06*		5.56
2-Aminobenzoic acid	138.00	120.10*	91.90	7.56
Xanthine	153.10	136.10*	135.10	5.84
Aminoadipic acid	162.10	98.00*	70.10	5.67
Glycylproline	173.09	70.07*	116.07	6.06
N-Acetylornithine	175.10	70.00*	115.30	5.01
Citrulline	176.10	159.10*		5.41
Isocitric acid	193.00	175.00*	156.00	6.06
5-Thymidylic acid	243.10	110.10*	127.10	4.90
Cytidine	244.09	112.05*	94.95	5.71
Uridine	245.20	113.00*	92.10	7.61
Adenosine	268.11	136.06*	119.04	6.09
Inosine	269.09	137.05*	110.04	8.50
Saccharopine	277.14	259.13*	231.13	10.40
Inosinic acid	349.06	137.05*	119.04	5.85

*quantitative ion

Table S3. Linearity, precision and recovery results of amino acids in embryo and larvae samples.

Amino acids	Calibration curves		Embryo				Larvae			
			Recovery (Mean n=7)			Precision (n=21)	Recovery (Mean n=7)			Precision (n=21)
	Equation	R^2	Low	Middle	High	RSD (%)	Low	Middle	High	RSD (%)
Phe	$y=2.01\times10^5$ x $+1.99\times10^6$	0.9992	114.0	98.7	112.0	4.83	102.0	96.0	102.0	4.75
Tyr	$y=5.76\times10^4$ x $+3.96\times10^4$	0.9977	89.1	98.2	104.0	5.60	96.4	105.0	98.2	14.51
Asp	$y=5.8\times10^3$ x $+1.62\times10^4$	0.9999	97.1	108.0	96.7	13.66	98.3	103.0	98.7	9.18
Glu	$y=7.11\times10^3$ x $+1.14\times10^4$	0.9990	87.8	107.2	94.3	7.15	102.0	97.0	101.0	3.98
His	$y=6.37\times10^4$ x $+4.05\times10^4$	0.9966	104.0	100.0	98.7	4.47	95.3	108.0	96.5	12.05
Cys	$y=4.61\times10^4$ x $+4.81\times10^3$	0.9996	99.9	110	95.3	6.55	98.0	103.0	98.5	9.88
Gln	$y=9.56\times10^4$ x -6.76×10^4	0.9995	95.6	103.5	101.0	4.36	102.0	96.6	101.0	7.77
Met	$y=1.86\times10^4$ x $+1.77\times10^4$	0.9997	98.2	82.7	109.0	6.03	92.4	101.0	100.0	10.24
Ile	$y=2.41\times10^3$ x $+8.11\times10^3$	0.9980	87.6	88.2	109.0	4.65	110.0	107.0	95.1	11.45
Lys	$y=9\times10^4$ x $+7.7\times10^5$	0.9908	99.5	103.0	98.5	11.39	95.2	99.5	102.3	12.80
Tau	$y=4.95\times10^3$ x $+3.36\times10^4$	0.9979	90.1	102.0	101.0	4.19	105.0	109.0	95.0	2.46
Asn	$y=9.88\times10^4$ x $+2.16\times10^4$	0.9995	99.6	90.5	105.0	6.48	106.0	95.6	101.0	5.47
GABA	$y=4.86\times10^4$ x $+5.45\times10^6$	0.9992	106.0	108.0	94.6	4.84	112.5	102.0	92.8	10.43
Pyr	$y=3.23\times10^3$ x $+1.87\times10^5$	0.9915	97.9	107.0	96.8	6.89	104.0	99.1	114.0	3.70
Ala	$y=1.07\times10^4$ x $+4.02\times10^4$	0.9903	112.0	106.0	94.8	11.26	108.0	112.0	93.1	5.31
Leu	$y=1.36\times10^5$ x $+1.97\times10^5$	0.9997	105.0	89.7	104.0	9.44	109.0	99.9	98.9	7.44
Ser	$y=1.68\times10^4$ x $+1.79\times10^5$	0.9980	98.9	118.0	91.5	5.95	113.0	103.0	96.1	7.39
Thr	$y=1.34\times10^4$ x $+4.15\times10^4$	0.9948	112.0	99.5	97.4	8.39	99.4	115.0	92.5	13.44
Arg	$y=6.56\times10^4$ x $+0.147$	0.9950	91.2	108.0	93.0	7.70	112.3	111.0	92.2	18.20
Val	$y=4.98\times10^4$ x $+0.0423$	0.9994	97.6	109.0	96.3	14.17	108.0	103.0	97.4	10.58
Pro	$y=9.53\times10^4$ x $+0.0798$	0.9996	103.0	106.0	96.4	9.12	110.0	99.3	99.1	12.62
Trp	$y=1.35\times10^5$ x $+2.13\times10^4$	0.9992	97.5	98.4	101.0	5.34	107.0	94.5	102.0	5.15
